# Comparison of [^18^F]DPA-814 with [^18^F]DPA-714 for TSPO Imaging in an Experimental Model

**DOI:** 10.1007/s11307-026-02094-9

**Published:** 2026-04-01

**Authors:** J. van der Bie, J. Bakker, E. J. Verschoor, E. Nutma, J. Middeldorp, W. Beaino, M. Kassiou, J. J. Danon, J. A. M. Langermans, A. D. Windhorst, M. A. Stammes

**Affiliations:** 1 Biomedical Primate Research Centre (BPRC), Lange Kleiweg 161, Rijswijk, 2288 GJ The Netherlands; 2 Department of Population Health Sciences, Unit Animals in Science and Society, Faculty of Veterinary Medicine, Utrecht University, Utrecht, The Netherlands; 3 Department Radiology & Nuclear Medicine, Amsterdam UMC Location Vrije Universiteit Amsterdam, De Boelelaan 1117, Amsterdam, 1081 HV The Netherlands; 4 Amsterdam Neuroscience, Brain Imaging, Amsterdam, The Netherlands; 5 School of Chemistry, Faculty of Science, University of Sydney, Sydney, NSW 2050 Australia

**Keywords:** DPA-714, PET-CT, SARS-CoV-2, Inflammation, Long covid, Macaque

## Abstract

**Purpose:**

[^18^F]DPA-714 is a valuable tracer for studying (neuro)inflammation, with well-characterized tracer kinetics and an established imaging window. However, its clinical utility is restricted by the TSPO polymorphism (rs6971), which influences binding affinity in humans. The newly developed tracer [^18^F]DPA-814 overcomes this limitation and has shown promising results in a preclinical rat model. To further assess its clinical potential, we compared [^18^F]DPA-814 to [^18^F]DPA-714 for inflammation imaging in SARS-CoV-2-infected macaques in a longitudinal setting.

**Procedures:**

Dynamic positron emission tomography (PET) imaging was conducted in four healthy macaques to identify the optimal imaging window for [^18^F]DPA-814. Four additional macaques were infected with SARS-CoV-2 and monitored for 12 months using whole-body PET-computed tomography (CT) with both tracers. Baseline scans were compared to PET-CTs obtained at 4, 9 and 16 days and at 6 and 12 months post-infection, covering the head, thorax and abdomen. Tracer uptake was assessed in several organs.

**Results:**

At baseline, [^18^F]DPA-814 showed higher lung uptake with minimal washout compared to [^18^F]DPA-714. Although lung lesions developed after infection, [^18^F]DPA-814 did not demonstrate lesion-specific uptake, unlike [^18^F]DPA-714. In the brain, the tracers also displayed divergent uptake patterns despite comparable TSPO levels across animals and regions.

**Conclusions:**

[^18^F]DPA-814 exhibits a distinct whole-body distribution, particularly in the lungs and brain, in both naïve and SARS-CoV-2-infected macaques compared with [^18^F]DPA-714, likely reflecting differences in tracer kinetics. Based on these data, [^18^F]DPA-814 may not fully replace [^18^F]DPA-714 for lung and brain imaging, and further studies are required to evaluate its suitability in other anatomical regions.

**Supplementary Information:**

The online version contains supplementary material available at 10.1007/s11307-026-02094-9.

## Introduction

Molecular imaging allows for minimal-invasive visualization of biological processes at both the cellular and molecular level, offering insights beyond traditional anatomical imaging. Among its various applications, inflammation imaging has gained particular interest as inflammation plays a central role in the pathophysiology of many diseases. A key molecular target for imaging inflammation is the outer mitochondrial membrane 18-kDa translocator protein (TSPO). TSPO is expressed throughout the whole body but is upregulated in activated microglia and systemic monocytes during inflammation [1]. TSPO can be visualized using PET-CT with the radiolabelled tracer *N,N*-diethyl-2-[4-(2-fluoroethoxy)phenyl]−5,7-dimethylpyrazolo[1,5-a]pyrimidine-3-acetamide ([^18^F]DPA-714) [2]. To date, [^18^F]DPA-714 has primarily been used to visualize inflammation in patients with neurodegenerative diseases such as multiple sclerosis (MS) and Alzheimer’s disease [3–6]. Recent studies indicate its potential for visualizing brain and pulmonary inflammation for instance after SARS-CoV-2 infection, highlighting its broader utility in infectious diseases [7–9].

Beyond acute inflammation in infectious disease research, [^18^F]DPA-714 has also been applied to investigate inflammatory processes associated with the long-term effects of SARS-CoV-2, commonly referred to as post-acute sequelae of COVID-19 (PASC) [10]. PASC, or long COVID, is defined by persistent or new symptoms lasting for several months after the initial SARS-CoV-2 infection [11]. PASC includes over 200 symptoms affecting multiple organ systems, thereby significantly impacting patients’ quality of life [12].

Despite the promising utility of [^18^F]DPA-714 for studying (neuro)inflammation in PASC, its main limitation is that its binding ability in humans depends on a single-nucleotide polymorphism at residue 147 (A147T) (rs6971) [13, 14]. The alanine-to-threonine substitution at this position reduces binding affinity and leads to interindividual variability in PET signal intensity, with approximately 23% of humans classified as low-affinity binder, 31% as mixed-affinity binders, and 46% as high-affinity binders. To overcome this limitation, a next-generation radiolabelled tracer, *N-*methyl-*N-*phenyl-2-[2-(4-(2-[^18^F]fluoroethoxy)phenyl)−5,7-dimethylpyrazolo[1,5-a]pyrimidine-3-acetamide ([^18^F]DPA-814), was developed [2]. In contrast to [^18^F]DPA-714, [^18^F]DPA-814 demonstrates equivalent affinity for both the wild-type and A147T variants of TSPO, effectively eliminating polymorphism-related differences in binding affinity. This was confirmed in cell-based assays and by autoradiography on post-mortem brain tissue of MS patients [2]. In addition, studies using a rat MS model revealed good brain uptake of [^18^F]DPA-814 with higher accumulation in inflamed regions compared to non-inflamed regions [2].

To further assess the clinical potential of [^18^F]DPA-814, SARS-CoV-2-infected macaques provide a valuable model for studying virus-induced inflammatory processes under controlled experimental conditions. Macaques closely resemble humans in both physiology and immune function, making them particularly suitable for translating preclinical findings [15]. Moreover, the use of larger animals such as macaques can improve the accuracy and translational relevance of imaging data, thereby complementing results obtained from earlier studies in smaller animals [16]. In the context of a SARS-CoV-2 infection, macaques have proven to be a suitable model for investigating the longitudinal course of infection, especially in the brain and lungs [8, 17, 18]. Collectively, these characteristics make SARS-CoV-2-infected macaques a good translational preclinical model for evaluating [^18^F]DPA-814 as a novel radiotracer for imaging inflammation [19, 20].

In this study, both [^18^F]DPA-714 and [^18^F]DPA-814 were employed to longitudinally assess inflammation in macaques following a SARS-CoV-2 infection. The aim of the study was to evaluate the two tracers in terms of whole-body distribution and to determine the potential of [^18^F]DPA-814 for clinical imaging applications.

## Material and Method

### Animals, Study Design and Ethics

Two groups of non-human primates (NHPs) from the Biomedical Primate Research Centre (BPRC) breeding colony were used in this study: one group (n = 4) for dynamic PET imaging with [^18^F]DPA-814 and another group (*n* = 4) for comparing [^18^F]DPA-814 to [^18^F]DPA-714 for whole body inflammation imaging following SARS-CoV-2 infection. These animals were part of a broader study on the immunological and pathological effects of SARS-CoV-2.

For dynamic PET imaging with [^18^F]DPA-814, two healthy naïve female rhesus macaques (*Macaca mulatta;* aged 17 and 20 year and weighing 7.5 and 9.8 kg) and two healthy naïve female cynomolgus macaques (*Macaca fascicularis;* aged 15 and 17 year and weighing 4.4 and 5.5 kg) were selected. For the comparison of [^18^F]DPA-814 with [^18^F]DPA-714 in SARS-CoV-2 infected animals, four healthy female rhesus macaques, aged 8–10 year and weighing 9.4–11.1 kg, were selected.

Prior to inclusion, all animals were confirmed healthy, based on physical examination by a veterinarian and evaluations of haematology and serum chemistry. They were socially housed in pairs and provided with a daily diet of commercial monkey pellets (Ssniff, Soest, Germany), supplemented with vegetables and fruit. Homemade and commercially available enrichment products were provided daily. Drinking water was available *ad libitum* through an automatic watering system. Animals were monitored at least twice daily for appetite, general behaviour and stool consistency. Animals designated for SARS-CoV-2 infection were transferred to a Biosafety Level 3 facility five weeks prior to infection for acclimatization. After infection, special attention was paid to clinical symptoms related to an airway infection such as sneezing, coughing, dyspnea, and breathing frequency. All necessary precautions were taken to ensure the animals’ welfare and minimize any discomfort.

**Fig. 1:**
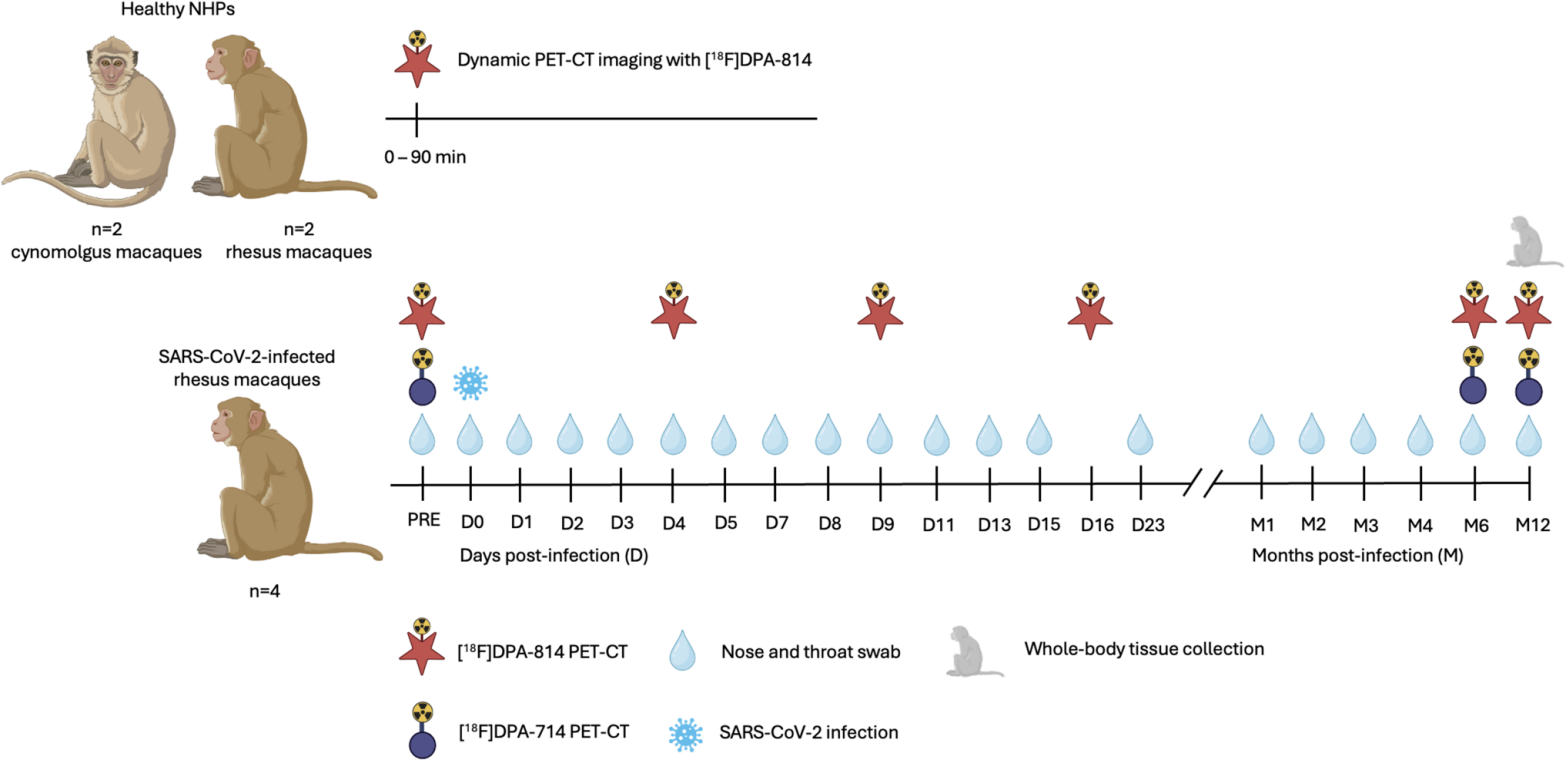
Study design. The study comprised two groups. The first group included two rhesus macaques and two cynomolgus macaques, which underwent 90-min dynamic PET imaging with [^18^F]DPA-814 (red radiotracer) following tracer injection. The second group included four rhesus macaques, which underwent multiple experimental procedures: [^18^F]DPA-814 PET-CT, [^18^F]DPA-714 PET-CT (purple radiotracer), nasal and tracheal swab collection (blue droplet), SARS-CoV-2 infection (virus), and whole-body tissue collection (grey rhesus macaque)

All experimental procedures, including intratracheal and intranasal infection, nasal and tracheal swab collections, PET-CTs, and dynamic PET imaging were conducted under sedation (Fig. 1). Animals were sedated via intramuscular injections of ketamine (10 mg/kg ketamine hydrochloride; Alfasan Nederland BV, Woerden, Netherlands) combined with medetomidine (0.05 mg/kg medetomidine hydrochloride; Sedastart; AST Farma B.V., Oudewater, Netherlands). After the procedures, upon the macaques’ return to their home cage, atipamezole (0.25 mg/kg atipamezole hydrochloride; Sedastop, ASTFarma B.V., Oudewater, Netherlands) was administrated intramuscularly to antagonize medetomidine.

All procedures, husbandry, and housing were performed in accordance with Dutch law and international ethical and scientific standards and guidelines (EU Directive 63/2010). The study involving SARS-CoV-2-infected animals was conducted as part of a larger study under project license AVD5020020209404 authorized by the national competent authorities under Dutch law, with additional approval from the institutional animal welfare body. BPRC is accredited by the American Association for Accreditation of Laboratory Animal Care (AAALAC) International.

### Virus Infection and Detection

Four rhesus macaques were infected with the SARS-CoV-2 strain UVE/SARS-CoV-2/2023/FR/GGR (Omicron variant, lineage EG.5.1.1, European Virus Archive) as part of a broader study. They were inoculated with a dose of 1 × 10^5^ TCID_50_ diluted in 5 ml PBS, administrated via combined intratracheal, just below the vocal cords, (4.5 ml) and intranasal (0.25 ml in each nostril) route. The detection of SARS-CoV-2 RNA in the swabs was conducted as previously described [17].

### PET-CT

#### Radiosynthesis of [^18^F]DPA-714

Radiosynthesis of [^18^F]DPA-714 was performed using previously described procedures [4]. [^18^F]DPA-714 was synthesized with a molar activity of 207 ± 28.6 GBq/μmol (mean ± SD), a radioactivity concentration of 233 ± 41.8 MBq/mL (mean ± SD), and a radiochemical purity of at least 98%.

#### Radiosynthesis of [^18^F]DPA-814

Radiosynthesis of [^18^F]DPA-814 was performed using previously described procedures [2]. [^18^F]DPA-814 was synthesized with a molar activity of 87 ± 29.8 GBq/μmol (mean ± SD), a radioactivity concentration of 205.6 ± 45.7 MBq/mL (mean ± SD), and a radiochemical purity above 95%.

#### Scan Acquisitions

Animals were fasted overnight before PET-CTs, in accordance with standard procedures [7, 8, 21, 22]. PET-CTs were obtained pre-infection to obtain a baseline value. PET-CTs were obtained using a MultiScan Large Field of View Extreme Resolution Research Imager (LFER) 150 PET-CT (Mediso Medical Imaging Systems Ltd., Budapest, Hungary) as described before [23]. Animals were positioned head-first supine with the arms up.

#### Static PET-CT Imaging

Following a scout-view, an intravenous bolus (1–2 ml) of 103 ± 27.3 MBq (mean ± SD) [^18^F]DPA-714 or 179 ± 27.2 MBq (mean ± SD) [^18^F]DPA-814 was administered. Three PET images of 10 min, each covering a single field of view (FOV) of the head, thorax and abdominal area, were obtained 10 min after injection, starting with the abdominal area, followed by the head and thorax region. Additionally, a CT of the three FOVs was acquired to use for anatomical information and attenuation correction. The main scan parameters applied for the CTs used in this manuscript were 80 kV, 720 μA and an exposure time of 0.09 s.

#### Dynamic PET Imaging

A CT scan was acquired just before tracer administration for attenuation correction. Each animal received an intravenous bolus (1–2 ml) of 128.3 ± 19.7 MBq (mean ± SD) [^18^F]DPA-814. A dynamic PET study was conducted for 90 min immediately following tracer administration, covering a single FOV of the head. The scans were acquired and rebinned into the following frame sequences: 10 s for first 3 min, 2 min for first 30 min, and 5 min for entire 90 min.

#### PET-CT Reconstruction

CT covering the thorax area was retrospectively gated in the expiration phase and afterwards reconstructed following a filtered back projection protocol with a RamLak filter and an isotropic voxel size of 321 μm [24]. The CTs covering the head and abdominal area were reconstructedμ following the same filtered back projection, RamLak filter and isotropic voxel size of 321μm. The emission data were iteratively reconstructed (OSEM3D, 8 iterations and 9 subsets with an isotropic voxel size of 0.8 mm) into a single frame PET image normalized and corrected for attenuation, scatter, and random coincidences using the CT, and corrected for radioactive decay.

#### PET-CT Analysis

PET-CT analysis was performed with VivoQuant 4.5 (Invicro, Boston, USA). Volumes of interest (VOIs) were manually delineated for representative areas of the liver and spleen with a size of at least 70 cm^3^ for the liver and 2.6 cm^3^ for the spleen. Whole brain VOIs were generated using an existing macaque brain template and adjusted for each animal based on individual size and shape. The necessary adjustments were based on both the PET and CT data. Lungs were defined via an automatic contouring tool on the thorax CT using a density range of −1000 to −400 Hounsfield units (HU), and lung lesions were identified using a density range of −400 to 1000 HU. Tracer uptake in the VOIs were quantified by the mean and peak standardized uptake values (SUVs).

### Necropsy and Tissue Sampling

The SARS-CoV-2-infected animals were sacrificed at 12 months post-infection to evaluate long-term pathological and immunological effects of infection as part of a broader study. Animals were anesthetized intravenously with a combination of ketamine (10 mg/kg) and medetomidine (0.5 mg/kg) and subsequently euthanized intravenously with Euthasol® (pentobarbital sodium and phenytoin sodium) (60 mg/kg). Necropsies were conducted following a standard protocol.

The brains were separated into two hemispheres as previously described for subsequent analyses, including immunofluorescent staining [7, 25]. The right hemisphere was fixed in 10% neutral buffered formalin for 72 h, then transferred to 0.1% PFA in PBS. The cerebrum, cerebellum and pons were subsequently dissected into 0.5 cm thick coronal parts anatomically dissected, processed and afterwards embedded in paraffin blocks. Multiple consecutive 5 μm sections were cut using a microtome (HistoCore MULTICUT R, Leica) for immunofluorescent staining.

### Immunofluorescence

Immunofluorescent staining for TSPO was performed on brain tissue sections of the pituitary gland, frontal cortex, and striatum. Sections were deparaffinized, rehydrated and subsequently quenched with 0.1% w/v glycine in PBS. For antigen retrieval, slides were incubated in citrate buffer (pH = 6.0) for 30 min at 98 °C. After cooling down and washing with PBS, slides were incubated in 3% v/v donkey serum (VWR) in PBS for 30 min at room temperature. Subsequently, slides were incubated overnight at 4 °C with the primary antibody rabbit-anti-TSPO (1:1000, Abcam, ab109497) diluted in universal antibody dilution buffer (U3510, Sigma-Aldrich). The slides were then washed with 0.05% v/v Tween-20 (Sigma-Aldrich) in PBS and incubated with the secondary antibody donkey-anti-rabbit (Alexa Fluor 594, IgG, Jackson ImmunoResearch, 711–585-152) diluted in universal antibody dilution buffer for 2 h at room temperature. After washing with 0.05% v/v Tween-20 (Sigma-Aldrich) in PBS, slides were incubated with Hoechst (1:2500) in PBS for 10 min at room temperature. Slides were washed with PBS and mounted with fluoromount G (SouthernBiotech; 0100–01). Fluorescent images were acquired using a whole slide scanner microscope (Olympus VS200). ZEN Microscopy software (Zeiss) was used for picture analyses.

## Results

### Whole-body Distribution of [^18^F]DPA-714 and [^18^F]DPA-814 in Naïve Animals

**Fig. 2:**
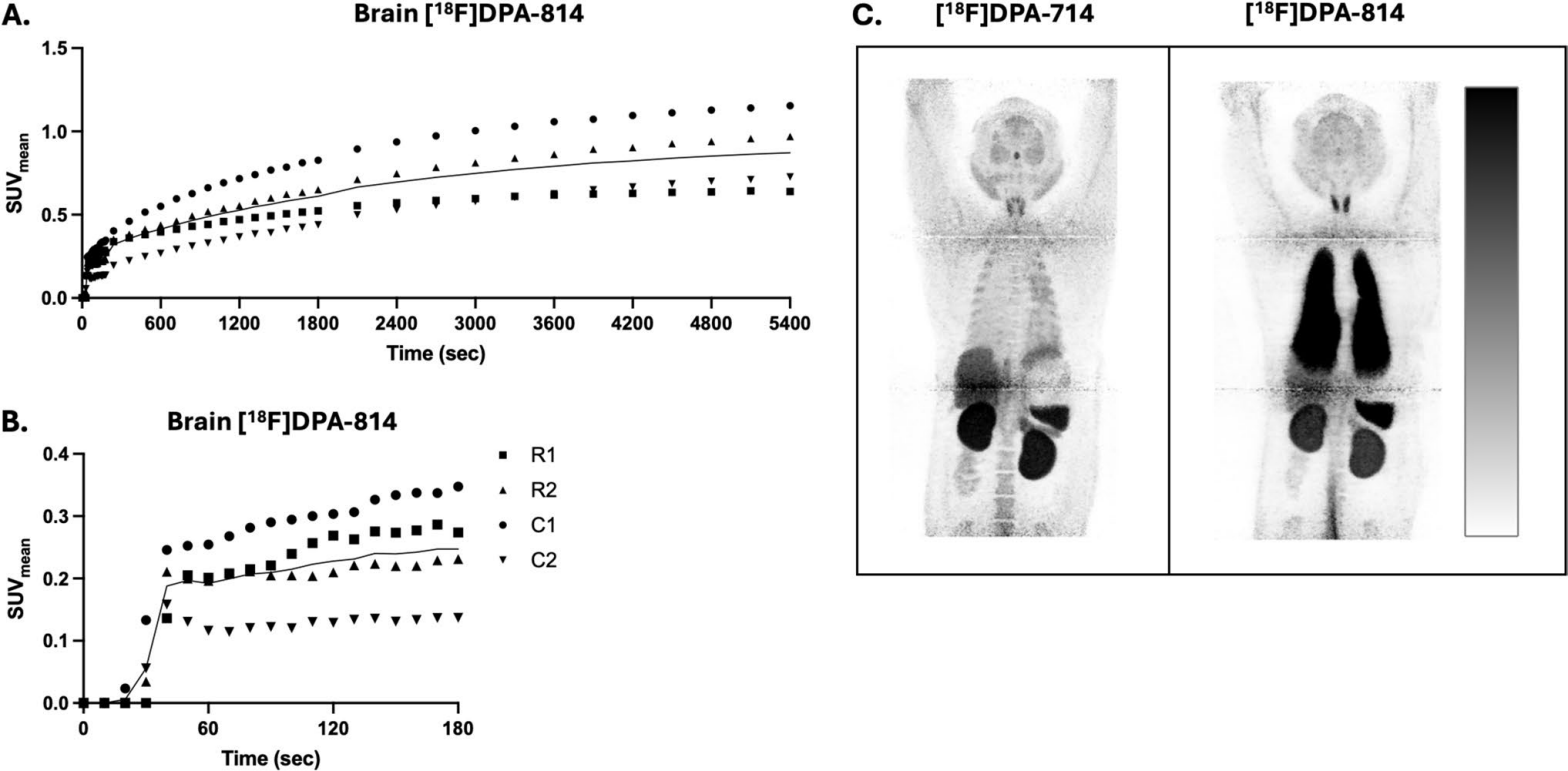
Comparison of [^18^F]DPA-714 and [^18^F]DPA-814 uptake in naïve animals. **A.** TACs of [^18^F]DPA-814 in the brain. **B.** Close-up view of [^18^F]DPA-814 TACs during the first 180 s. Each symbol represents an individual animal, and the line denotes the mean value of all animals. R1 and R2 correspond to rhesus macaques, and C1 and C2 to cynomolgus macaques. **C.** Combined whole-body maximum intensity projections (MIPs; 29.6 mm thickness) of three FOVs (head, thorax and abdomen) for [^18^F]DPA-714 and [^18^F]DPA-814 in the same animal. Data and window-level settings are synchronised

[^18^F]DPA-714 is a valuable tracer for studying (neuro)inflammation in NHPs, with its tracer kinetics and optimal imaging window well characterized [2, 8, 9, 26, 27]. In contrast, data on the tracer kinetics and the ideal imaging time frame for [^18^F]DPA-814 in NHPs are currently lacking. To address this, dynamic head PET imaging with [^18^F]DPA-814 was performed in naïve animals. The resulting brain time activity curves (TACs; expressed in SUV_mean_) of [^18^F]DPA-814 for all animals are shown in Fig. 2A and B. [^18^F]DPA-814 showed fast initial uptake in the brain, followed by a gradual increase during the 90-min imaging period, with comparable uptake in rhesus and cynomolgus macaques. No clear plateau or washout phase of the tracer was observed. Despite the absence of a well-defined plateau, follow-up scans were acquired as static PET-CTs starting 10 min after tracer injection, due to greater inter-animal variability at later time points.

To establish baseline values and assess whether [^18^F]DPA-814 produces a comparable whole-body distribution to [^18^F]DPA-714, four animals were injected with [^18^F]DPA-714 and [^18^F]DPA-814 on consecutive days before SARS-CoV-2 infection. Whole-body PET-CTs showed notable differences in tracer uptake patterns between the two tracers in all animals (Fig. 2C and Supp. Figure 1). [^18^F]DPA-714 demonstrated diffuse uptake in the lungs and visible uptake in the salivary glands and spine. In contrast, [^18^F]DPA-814 showed higher uptake in the lungs and cervical lymph nodes, with lower background uptake throughout the entire body. Despite these differences, both tracers demonstrated similar visualization patterns with increased uptake in the same lung regions (Supp. Figure 2) and detectable uptake in the brain, liver, spleen, and kidneys (Fig. 2C). Overall, [^18^F]DPA-714 and [^18^F]DPA-814 showed distinct whole-body distribution patterns.

### Pulmonary Distribution of [^18^F]DPA-814 During the Acute Phase of SARS-CoV-2 Infection

**Fig. 3:**
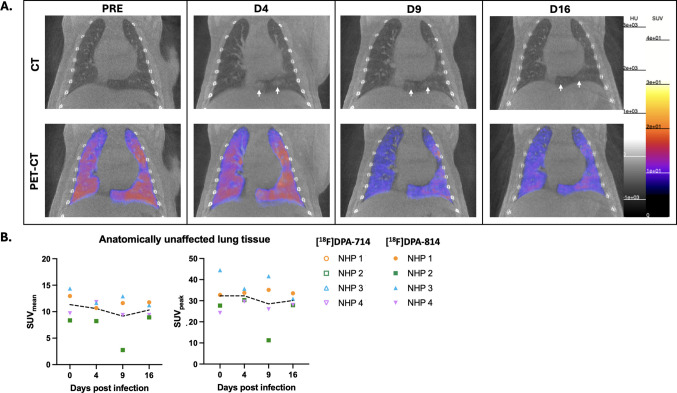
High pulmonary uptake of [^18^F]DPA-814 without clear visualization of SARS-CoV-2-induced lesions or inflammation. **A.** Representative coronal (PET-)CT slices of the development of pulmonary lesions in a macaque. Window-level settings are synchronised. White arrows indicate SARS-CoV-2-induced lesions. **B.** Development of the SUV_mean_ and SUV_peak_ in the anatomically unaffected lung tissue over time in the acute phase after SARS-CoV-2 infection. Each symbol represents an individual animal, and the dotted line indicates the average SUV_mean_ or SUV_peak_

Given that [^18^F]DPA-714 has demonstrated its utility in visualizing SARS-CoV-2-induced pulmonary lesions and inflammation in the acute phase [8], we aimed to evaluate [^18^F]DPA-814 as a potential new radiolabelled tracer in the same experimental model. PET-CT imaging with [^18^F]DPA-814 was performed before SARS-CoV-2 infection and on day 4, 9 and 16 after infection to study its ability to visualize lung lesions and inflammation of the mediastinal lymph nodes during the acute phase of infection (Fig. 3A and Supp. Figure 3). Following exposure with SARS-CoV-2, active replicating virus was detected in all animals up to day 9 post-infection through the presence of subgenomic messenger RNA in the nasal and tracheal swabs of all animals. In addition, chest CTs revealed pulmonary lesions in all animals after infection (Supp. Figure 3). However, PET analysis showed no specific uptake of [^18^F]DPA-814 in the lung lesions over time, unlike the uptake observed with [^18^F]DPA-14 (Supp. Figure 3) [8]. Previously, in the lung tissue surrounding the lung lesions, the anatomically unaffected lung tissue, an increase in [^18^F]DPA-714 uptake was detected after infection [8]. In contrast, [^18^F]DPA-814 showed no increase in uptake anatomically unaffected lung tissue, as both average SUV_mean_ (PRE: 11.3, D4: 10.6, D9: 9.2, D16: 10.3) and SUV_peak_ (PRE: 32.3, D4: 32.3, D9: 28.5, D16: 30.1) remained relatively consistent throughout the entire observation period with a similar pattern observed in all four animals (Fig. 3B). Consistent with the findings in Fig. 2C, [^18^F]DPA-814 exhibited high uptake throughout the lung before and after infection, limiting its ability to distinguish between unaffected and affected pulmonary tissue. Nevertheless, although differences in overall uptake, the uptake pattern in anatomically unaffected lung tissue remained similar between the [^18^F]DPA-814 PET-CTs acquired before and after infection on D4, D9 and D16, which is particularly noticeable because the uptake is not uniform. Despite these differences, [^18^F]DPA-714 and [^18^F]DPA-814 displayed similar visualization patterns with increased uptake in the same lung regions (Supp. Figure 2).

### Brain Uptake of [^18^F]DPA-714 and [^18^F]DPA-814 at 6 and 12 Months after SARS-CoV-2 Infection

**Fig. 4:**
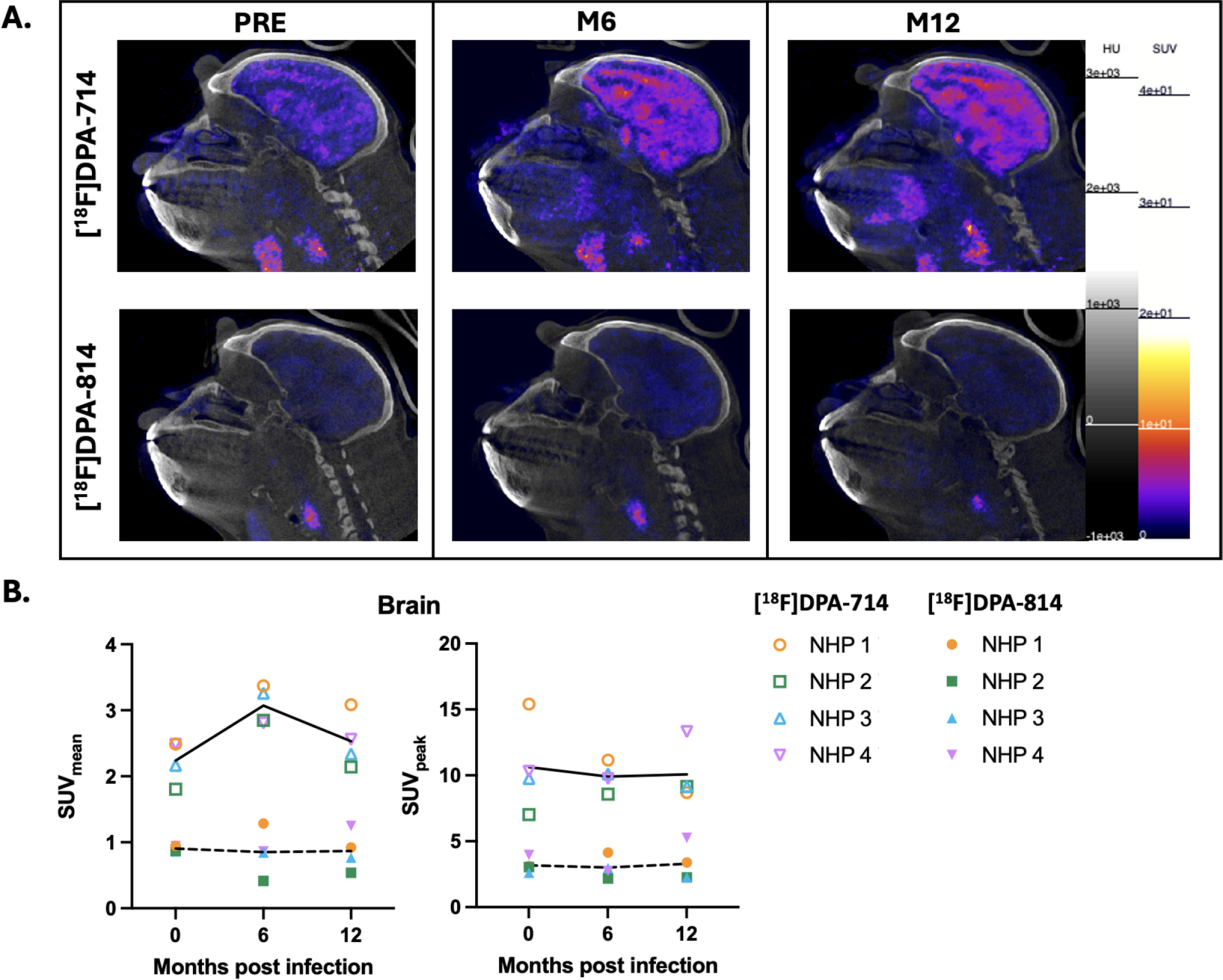
Increased [^18^F]DPA-714 uptake in the brain following SARS-CoV-2 infection. **A.** Representative sagittal brain PET-CT slices from a macaque. Window-level settings are synchronised. **B.** SUV_mean_ and SUV_peak_ values in the whole brain over time during the post-acute phase of SARS-CoV-2 infection. Each symbol indicates an individual animal; open symbols correspond to [^18^F]DPA-714 imaging and filled symbols to [^18^F]DPA-814. The continuous line represents the SUV_mean_ or SUV_peak_ average of [^18^F]DPA-714, while the dotted line shows the average of [^18^F]DPA-814

Building on previous findings demonstrating the application of [^18^F]DPA-714 in detecting SARS-CoV-2-associated neuroinflammation in the post-acute phase [7], we explored whether [^18^F]DPA-814 could visualize neuroinflammatory processes during these later phases of infection as well. For this, scans with both tracers were obtained on consecutive days at 6 and 12 months after infection (Fig. 4A and Supp. Figure 4). Based on average SUV_mean_ values, PET-CTs with [^18^F]DPA-714 revealed an increased tracer uptake across the brain after SARS-CoV-2 infection in all animals (Fig. 4B). In all animals the tracer uptake peaked at 6 months post-infection (average SUV_mean_: 3.1, increase of 37.1%), while the uptake at 12 months post-infection remained above the pre-infection levels (average SUV_mean_: 2.5, increase of 12.9%). In contrast, [^18^F]DPA-814 showed no increase in brain tracer uptake over the course of infection (Fig. 4B). The tracer uptake remained relatively consistent with a similar pattern observed in all four animals (average SUV_mean_: PRE: 3.2, M6: 3.0, M12: 3.3). In addition, [^18^F]DPA-814 uptake was substantially lower than [^18^F]DPA-714 uptake. For both tracers, the average SUV_peak_ remained relatively stable ([^18^F]DPA-714 PRE: 10.6, M6: 9.9, M12: 10.1; [^18^F]DPA-814 PRE: 3.2, M6: 3.0, M12: 3.3), with no substantial differences observed across all animals.

### *Ex vivo* Validation of TSPO Expression in the Pituitary Gland, Frontal Cortex and Striatum After a SARS-CoV-2 Infection

**Fig. 5:**
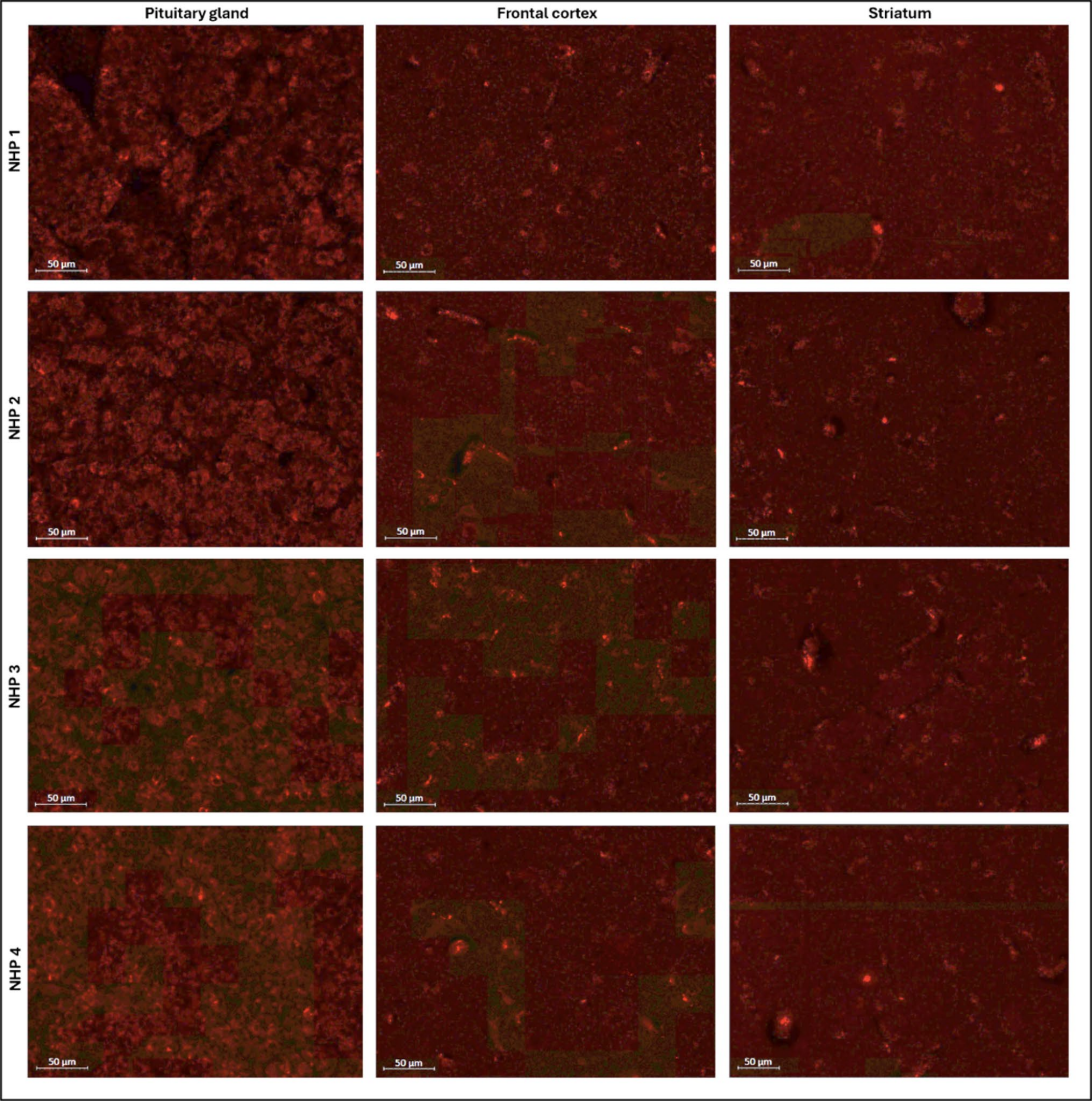
Comparable levels of TSPO expression in the pituitary gland, frontal cortex, and striatum of all SARS-CoV-2 infected macaques

Given the observed differences in brain tracer uptake between [^18^F]DPA-714 and [^18^F]DPA-814, we aimed to confirm the presence of TSPO in the brains of the infected animals. Therefore, we performed immunofluorescent staining on postmortem brain tissue to assess TSPO expression. Analyses focused on the pituitary gland, frontal cortex, and striatum, as previous results from SARS-CoV-2 infected macaques and individuals with PASC showed that these regions are highly likely affected [25, 28]. Visual assessment showed that TSPO expression was generally consistent across all animals (Fig. 5). Noticeable differences in expression were observed between the three brain regions. However, these patterns remained consistent across all NHPs, indicating that the *in vivo* differences in tracer uptake are not due to variations in TSPO expression.

### Spleen, Liver and Kidney Distribution of [^18^F]DPA-714 and [^18^F]DPA-814 at 6 and 12 Months After a SARS-CoV-2 Infection

**Fig. 6:**
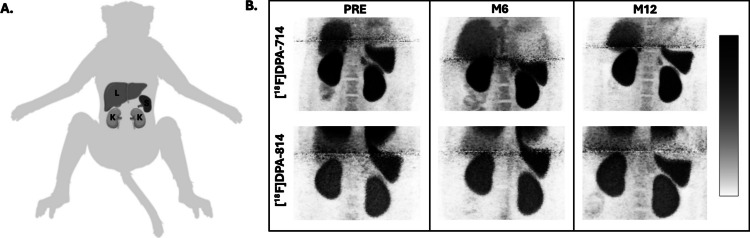
Comparison of [^18^F]DPA-714 and [^18^F]DPA-814 uptake in the spleen, liver and kidneys after SARS-CoV-2 infection. **A.** Schematic illustration showing the anatomical positions of the spleen (S), liver (L) and kidneys (K) in a macaque. **B.** Combined whole-body MIPs (29.6 mm thickness) of two FOVs (thorax and abdomen) for [^18^F]DPA-714 and [^18^F]DPA-814 in the same animal. Data and window-level settings are synchronised. PET imaging was performed 10 min post injection

Considering the spleen’s role as a key secondary lymphoid organ, we investigated [^18^F]DPA-714 and [^18^F]DPA-814 uptake in the spleen at 6 and 12 months post-SARS-CoV-2 infection. Both tracers showed relatively high spleen uptake with no substantial differences between pre- and post-infection (Fig. 6 and Supp. Figure 5). Liver and kidney uptake were also evaluated, given their role in clearing lipophilic small-molecule radiotracers. Uptake of both tracers was observed in the liver and kidneys with no substantial differences between pre- and post-infection (Fig. 6). These results show that both radiotracers visualize myeloid cells in the spleen and are cleared via the liver and kidneys, with uptake remaining stable from pre- to post-infection.

## Discussion

This study primarily aimed to assess [^18^F]DPA-814 as a novel radiotracer for imaging inflammation and to determine whether its spatial distribution parallels that of [^18^F]DPA-714, thus providing evidence for its suitability in clinical translation. To this end, we compared the distribution of [^18^F]DPA-814 to [^18^F]DPA-714 in naïve animals to evaluate tracer kinetics and in SARS-CoV-2-infected animals to assess the ability of [^18^F]DPA-814 to detect inflammation.

Our results show that the whole-body distribution of [^18^F]DPA-814 differs from that of [^18^F]DPA-714 in both naïve and infected animals. In naïve animals, the most striking difference was observed in the lungs, where [^18^F]DPA-814 exhibited higher overall uptake than [^18^F]DPA-714. Despite this difference, both tracers displayed similar visualization patterns, with increased uptake in the same lung regions. However, the overall high pulmonary uptake of [^18^F]DPA-814 hinders the detection of localized changes.

During the acute phase of infection with SARS-CoV-2, pulmonary lesions were observed. However, these lesions showed no specific [^18^F]DPA-814 uptake, and only minimal differences were observed in anatomically unaffected lung tissue. This is in contrast to what has been observed before with [^18^F]DPA-714 where we detected specific tracer uptake in the lesions with evidence of inflammatory processes in anatomically unaffected lung tissue [8]. The difference in tracer uptake is likely attributable to the high pulmonary uptake of [^18^F]DPA-814 with minimal washout. This was already seen in naïve animals and limits the ability to distinguish between affected and unaffected pulmonary tissue. The higher lung persistence of [^18^F]DPA-814, which was also observed in a rat MS model [2], may be related to its increased lipophilicity compared to [^18^F]DPA-714.

During the post-acute phase, PET-CTs with [^18^F]DPA-714 revealed increased tracer uptake across the brain similar to what has been observed previously after SARS-CoV-2 infection [7], whereas [^18^F]DPA-814 showed no such increase. Additionally, overall brain uptake of [^18^F]DPA-814 was lower than that of [^18^F]DPA-714. This contrast may be attributed to the higher affinity of [^18^F]DPA-814 for TSPO, which could reduce plasma availability by promoting faster, more extensive binding to TSPO expressed on peripheral immune cells, as previously suggested by Beaino et al*.* [2]. The elevated lung uptake of [^18^F]DPA-814 might further amplify this effect, resulting in even lower plasma tracer levels and reduced brain uptake. However, given the rapid blood clearance of both tracers, differences in blood half-life are unlikely to account for the observed findings. Instead, the higher lipophilicity of [^18^F]DPA-814 may contribute substantially by promoting increased nonspecific binding and reduced washout, thereby limiting contrast generation for neuroinflammation imaging. Blood–brain barrier permeability might also contribute, as a slightly lower peak brain uptake was observed for [^18^F]DPA-814 compared to [^18^F]DPA-714, but this alone cannot account for the observed differences. Immunohistochemical staining of the pituitary gland, frontal cortex, and striatum revealed comparable TSPO expression in the animals, indicating that the observed differences in tracer uptake arise from tracer-specific properties rather than biological factors.

The immune system is known to play a critical role in the response to SARS-CoV-2, as shown, for instance, by Meijer et al*.,* who reported elevated levels of dendritic cells up to 30 days post-infection [8]. To further investigate immune involvement, we assessed [^18^F]DPA-714 and [^18^F]DPA-814 uptake in the spleen, a key secondary lymphoid organ. Both tracers showed relatively high spleen uptake with no substantial changes between pre- and post-infection. Liver and kidney uptake, reflecting clearance of lipophilic small-molecule radiotracers, was also observed without notable differences over time, indicating consistent hepatic and renal clearance.

As noted previously [8], it remains unclear whether the rs6971 polymorphism occurs in rhesus macaques, which could affect the suitability of this model for evaluating [^18^F]DPA-814. In humans, the rs6971 polymorphism influences tracer binding affinity, and its presence or absence in macaques could impact tracer uptake and signal interpretation. However, the low variability among animals in our study and the comparison of pre-infection scans with multiple post-infection scans suggest that any potential effects of this polymorphism are minimal. Additionally, the consistent uptake patterns across animals support the reliability of our results, indicating that [^18^F]DPA-814 imaging in macaques can still provide meaningful insights into tracer kinetics and visualization patterns despite uncertainty regarding the polymorphism.

A possible limitation of this study could be the limited number of included animals. However, two to four NHPs are generally considered sufficient for the evaluation of new PET-tracers, as performed in this proof-of-principle study. In addition, the inclusion of multiple longitudinal scans per animal over an extended period, together with the limited variation between animals, supports the reliability of the findings.

In conclusion, [^18^F]DPA-814 exhibits a different whole-body distribution in both naïve and SARS-CoV-2-infected animals compared to [^18^F]DPA-714. These differences limit the detection of localized changes and the distinction between affected and unaffected tissues. Therefore, based on current findings, while [^18^F]DPA-814 is not affected by the polymorphism, this next-generation radiotracer might not fully achieve the intended role of replacing [^18^F]DPA-714 for lung and brain imaging applications. Additional studies are required to determine its suitability in other anatomical regions.

## Supplementary Information

Below is the link to the electronic supplementary material.

ESM 1 (DOCX 4.48 MB)

## Data Availability

The data acquired and analysed during the current study are available from the corresponding author on reasonable requests.

## Abbreviations

AAALAC: American Association for Accreditation of Laboratory Animal Care
BPRC: Biomedical Primate Research Centre
CT: Computed tomography
FOV: Field of view
HU: Hounsfield unit
LFER: Large Field of View Extreme Resolution Research Imager
MIP: Maximum intensity projections
MS: Multiple sclerosis
NHP: Non-human primate
PASC: Post-acute sequelae of COVID-19
PET: Positron emission tomography
SUV: Standardized uptake value
TAC: Time activity curve
TSPO: 18-KDa translocator protein
VOI: Volume of interest
