# Supplementary material for: Comparison of [18F]DPA-814 with [18F]DPA-714 for TSPO Imaging in an Experimental Model
Source: Mol Imaging Biol. 2026 Apr 1;28(3):502–12. doi: 10.1007/s11307-026-02094-9 (PMC13337680; doi:10.1007/s11307-026-02094-9)
Supplement: Supplementary file 1 — (DOCX 4.48 MB) [file 11307_2026_2094_MOESM1_ESM.docx]

Supplementary data belonging to:

**Comparison of [^18^F]DPA-814 with [^18^F]DPA-714 for TSPO imaging in an experimental model**

J. van der Bie^1,2^, J. Bakker^1^, E. J. Verschoor^1^, E. Nutma^1^, J. Middeldorp^1^, W. Beaino^3,4^, M. Kassiou^5^, J.J. Danon^5^, J.A.M. Langermans^1,2^, A.D. Windhorst^3,4^, M.A. Stammes^1^


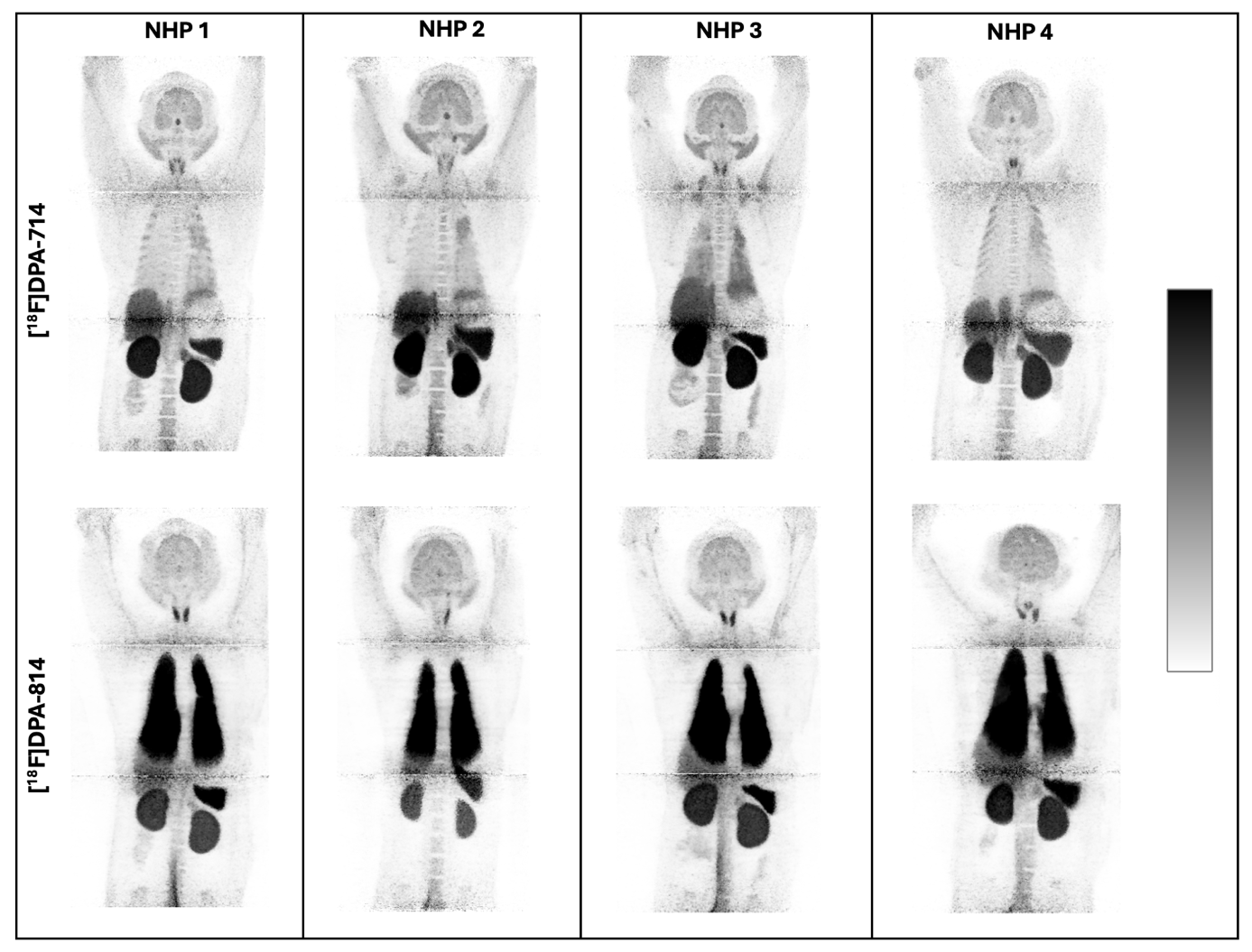


***Supplementary Fig. 1:*** *Comparison of [^18^F]DPA-714 and [^18^F]DPA-814 uptake in naïve animals. Combined whole-body MIPs (29.6 mm thickness) of three FOVs (head, thorax and abdomen) for [^18^F]DPA-714 and [^18^F]DPA-814 acquired in the same animal across all four animals. Data and window-level settings are synchronised.*


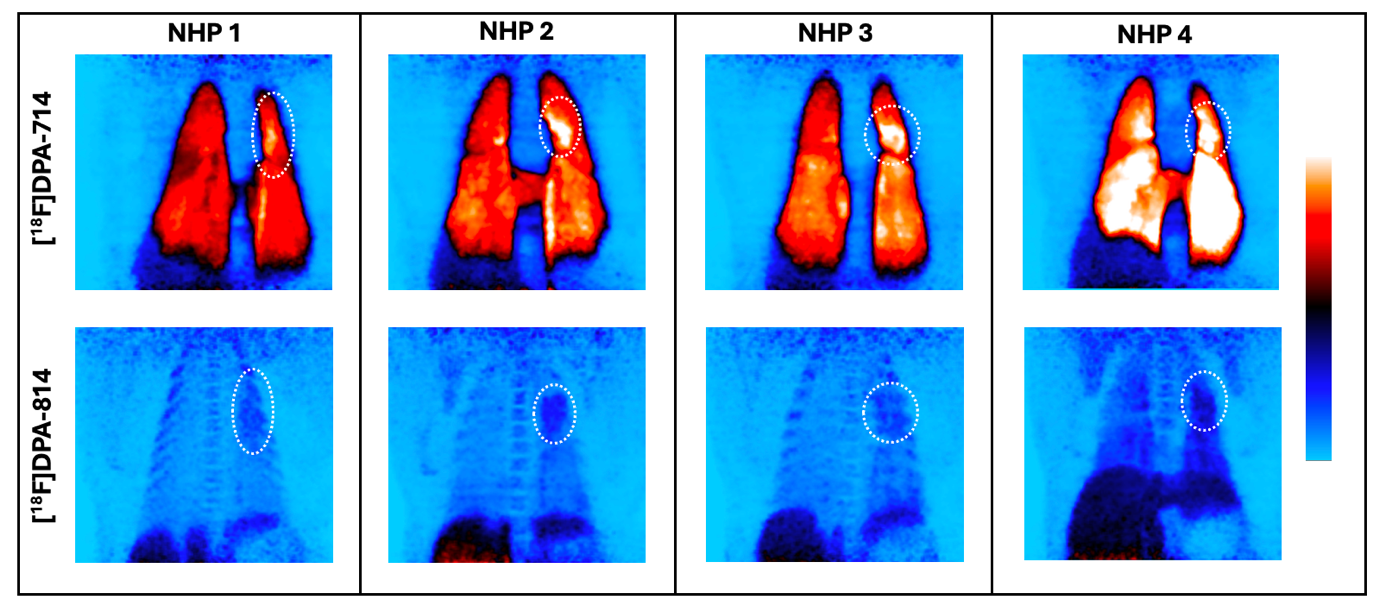


***Supplementary Fig. 2:*** *Increased uptake of [^18^F]DPA-714 and [^18^F]DPA-814 in the same lung region of naïve animals. Combined whole-body MIPs (29.6 mm thickness) of the thoracic FOV are shown for [^18^F]DPA-714 and [^18^F]DPA-814, acquired in the same animal across all four animals. Data and window-level settings are synchronised between the scans but not between the tracers. The signal in the [^18^F]DPA-714 had to be increased to determine the similarities in uptake over the different lung regions between the two tracers. The discontinuous circle indicates the lung region showing increased uptake for both tracers.*


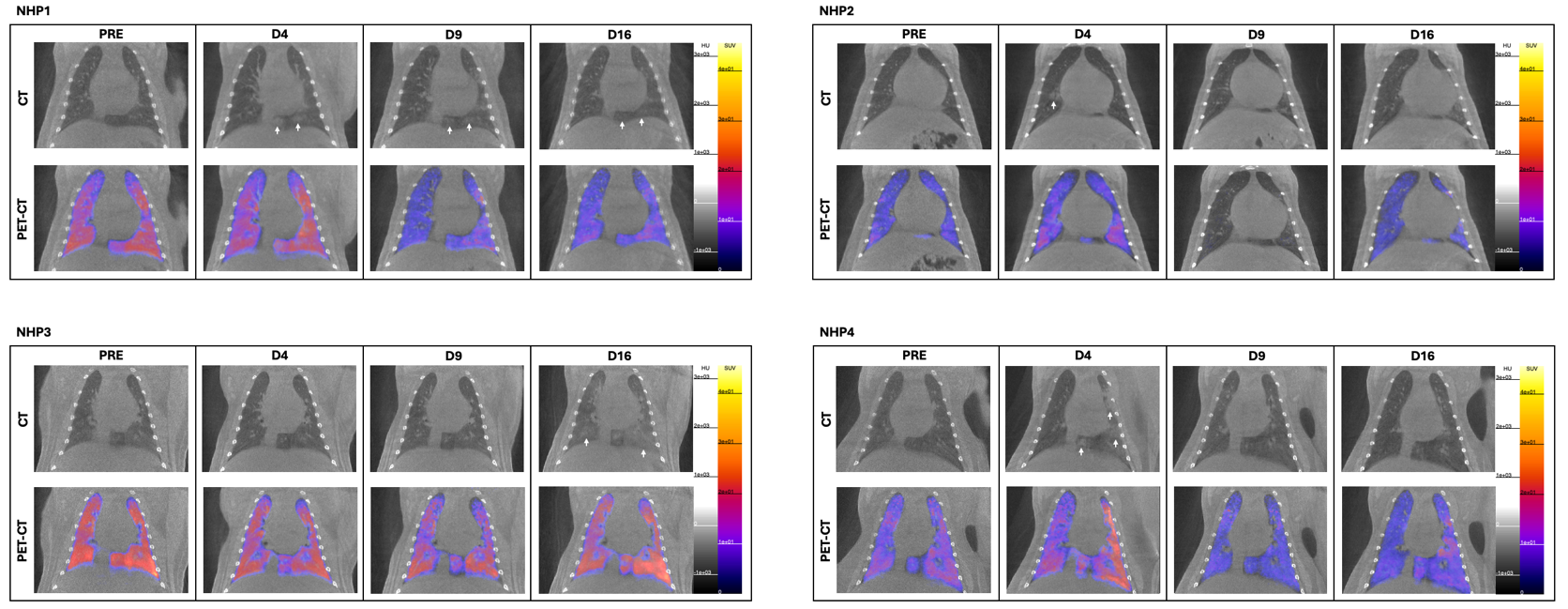


***Supplementary Fig. 3:*** *High pulmonary uptake of [^18^F]DPA-814 without clear visualization of SARS-CoV-2-induced lesions or inflammation. Representative coronal (PET-)CT slices of the development of pulmonary lesions across all four animals. Window-level settings are synchronised. White arrows indicate SARS-CoV-2-induced lesions.*


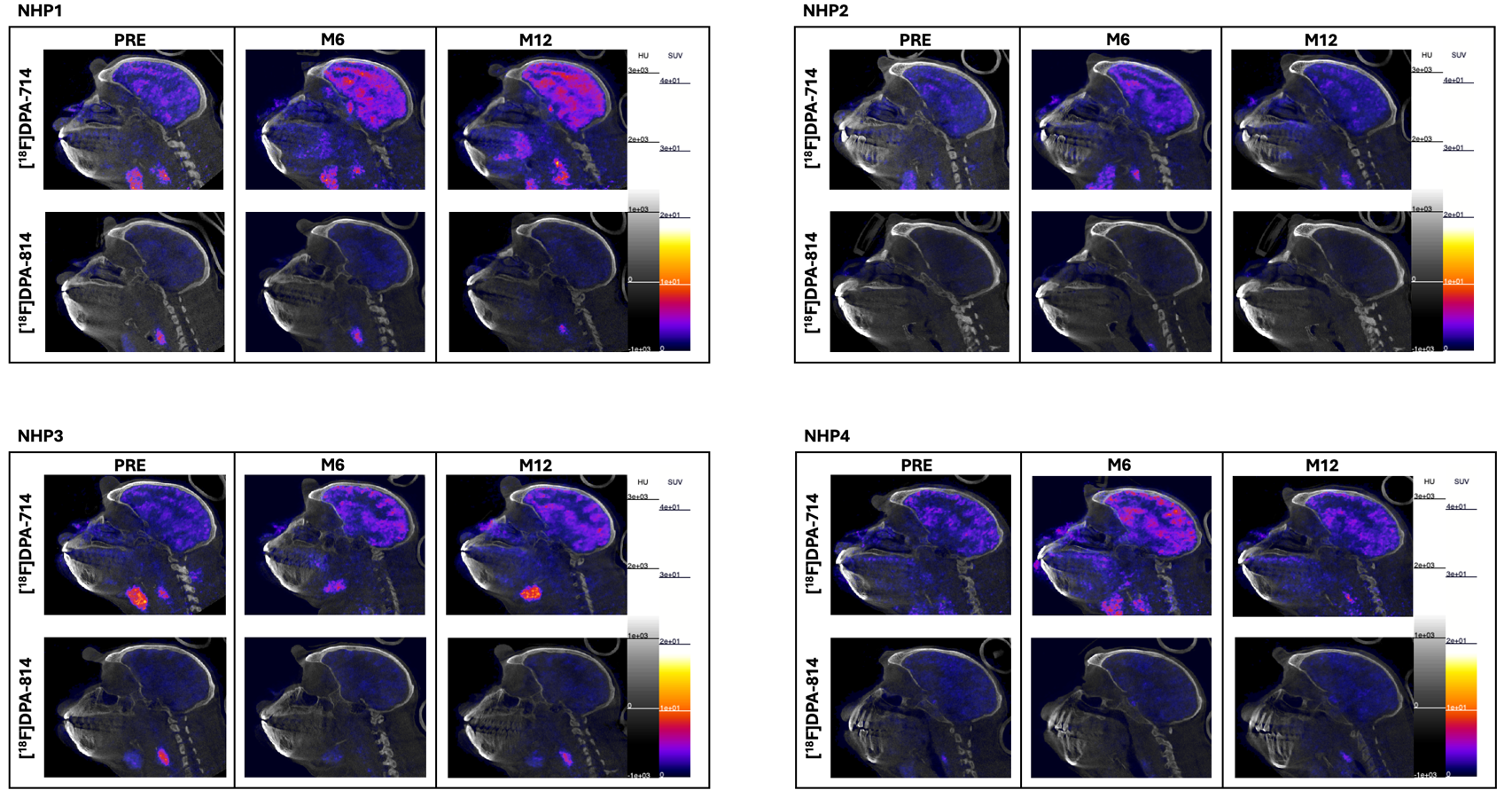


***Supplementary Fig. 4:*** *Increased [^18^F]DPA-714 uptake in the brain following SARS-CoV-2 infection. Representative sagittal brain PET-CT slices of all four animals. Window-level settings are synchronised.*


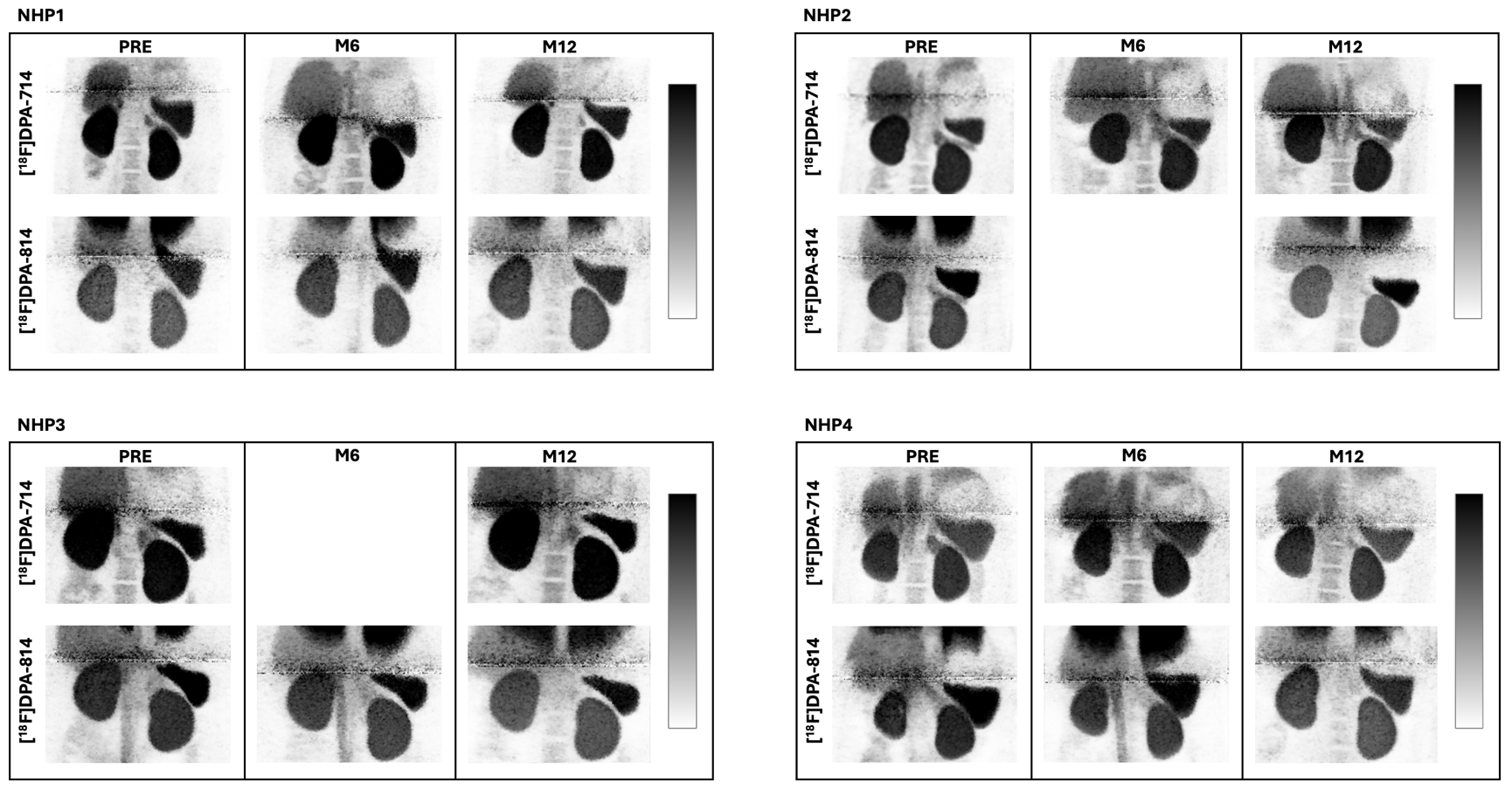


***Supplementary Fig. 5:*** *Comparison of [^18^F]DPA-714 and [^18^F]DPA-814 uptake in the spleen, liver and kidneys after SARS-CoV-2 infection.* *Combined whole-body MIPs (29.6 mm thickness) of two FOVs (thorax and abdomen) for [^18^F]DPA-714 and [^18^F]DPA-814 acquired in the same animal across all four animals. Data and window-level settings are synchronised. Images of PET scans from NHP2 and NHP3 at 6 months post-infection were not obtained due to limited availability of radioactivity.*
